# Mental health and perceived social support among nurses working in primary care

**DOI:** 10.3389/fpubh.2025.1694797

**Published:** 2025-12-04

**Authors:** Mariola Pietrzak, Ewa Kobos, Iwona Kiersnowska, Karolina Prasek, Edyta Krzych-Fałta, Zofia Sienkiewicz, Tomasz Kryczka

**Affiliations:** 1 Department of Development of Nursing, Social and Medical Sciences, Faculty of Health Sciences, Medical University of Warsaw, Warsaw, Poland; 2 Department of Nursing Propedeutics, Faculty of Health Sciences, Medical University of Warsaw, Warsaw, Poland

**Keywords:** mental health, social support, nurse practitioner, anxiety, depression

## Abstract

**Introduction:**

Primary care is a critical component of healthcare systems worldwide, and the way it is organized varies from country to country. Nursing staff are essential to patient care. The quality of patient care depends on the organization of healthcare services and the physical and mental well-being of healthcare professionals. The latter may be related to perceived social support. The aim of the study was to assess the prevalence of anxiety and depressive symptoms, and to analyse perceived social support among nursing staff working in primary care clinics.

**Methods:**

A cross-sectional study was conducted from July 2022 to January 2023 among nurses working in a network of private primary care outpatient clinics. The Polish adaptation of the Multidimensional Scale of Perceived Social Support (MSPSS) measured perceived social support, and a modified Hospital Anxiety and Depression Scale (HADS) assessed the mental well-being of the nurses. Date were analysed using Statistica 13.3, with a statistical significance level set at 0.05.

**Results:**

The study involved 175 nurses working in outpatient clinics of a privately-owned healthcare group. The results revealed that the nursing staff had symptoms of mental health disorders, such as anxiety (21.71%), depression (8%), and irritability (47.43%). In total, the respondents scored 15.54 ± 9.02 points on the HADS-M scale. The nursing staff rated perceived social support at 69.79 ± 13.08 points. Nurses in managerial positions reported higher overall perceived social support (*p* = 0.042). A negative correlation was found between the age of nursing staff and perceived support from significant others (*p* = 0.039), as well as between the number of workplaces and perceived support from family members (*p* < 0.01).

**Conclusion:**

Nurses who scored higher on mental well-being also rated their perceived social support higher. Further research is needed to explore how perceived social support from key individuals in the workplace affects the mental health of nurses employed in primary care.

## 1 Introduction

Nurses play a vital role in healthcare, contributing to improved patient outcomes (1). In Poland, 17.4% of registered nurses work in primary health care (PHC) (2). PHC is an essential component of the healthcare system in Poland, where services are provided free of charge to all citizens and financed by the National Health Fund (NHF) (3). However, patients may purchase optional healthcare packages of a similar scope from private providers, whose services are not covered by the NHF.

Regardless of their employment status — in private or public healthcare facilities — nurses provide a wide range of primary care services with a focus on health promotion and disease prevention (4). They empower individuals, families, and communities to care for their own health (5).

According to the WHO, well-being and mental health are basic human rights (6, 7). Expanding the scope of primary care nurse practice (5, 8) entails greater responsibility for patient care, potentially increasing nursing staff exposure to stress. This exposure may impact well-being and work performance (9–12). Nurses are among the professions most vulnerable to anxiety and stress (13). The challenges associated with meeting growing expectations and professional demands may raise concerns about nurses’ working conditions and their ability to cope with stress-inducing factors. Evidence from the limited studies conducted before the COVID-19 pandemic suggests that primary care nurses already had high levels of emotional exhaustion (23–31%) and were at risk of burnout, anxiety, and depression (14, 15). During the COVID-19 pandemic, these issues intensified, with anxiety (68.1%) and depression (55.6%) reported as common among primary healthcare professionals (16).

Stressors are constantly present in their work setting (17) and may negatively impact their overall well-being, potentially leading to anxiety and depressive symptoms (15, 18). In a study conducted in Poland (19), low level of life satisfaction was more frequently reported by nurses working in primary care (34.3%) and outpatient specialist care (31.9%) compared with hospital nurses (22.5%). Studies on anxiety and depression levels were conducted primarily among hospital nurses during the COVID-19 pandemic. The prevalence of depressive symptoms ranging from mild to severe among Polish nurses was reported at 62.98% in the study by Rachubińska et al. (20), compared with 35% among Chinese nurses (21), 34.7% among Iranian nurses (22), and 30% among Australian nurses (17). The prevalence of anxiety was reported at 43.4% among Iranian nurses (22), 41.2% among Australian nurses (17), and 65.7% among Polish nurses (20). However, it is difficult to estimate the scale of this phenomenon due to differences in how such disorders are reported in different countries (23).

A synthesis of available research on interventions aimed at improving the mental health of primary care nurses showed that none of the seven reviewed studies focused exclusively on nurses employed in primary care clinics, and none assessed outcomes such as anxiety or depression symptoms (24). Similarly, another systematic review found limited evidence that staff-focused interventions reduce occupational burnout or stress (25).

Social support is an important and potentially significant predictor of well-being and mental health (26, 27). Social support is defined as the assistance and protection provided by others. Support can be formal, such as from immediate superiors, or informal, such as from family and colleagues (28). Support can be assessed from two perspectives: organizational and social. Evidence suggests that people in more individualistic Western cultures, which emphasize personal responsibility for well-being, seek social support more often than those in collectivist cultures, such as Asian societies (29).

When facing difficulty, threat, or illness, a person seeks out specific types of support according to their needs, drawing from available sources in their environment. These social networks include sources of support such as family, friends, and neighbors. Other sources may include colleagues, supervisors, religious groups, community organizations, support groups, and professionals trained to provide assistance, such as therapists and physicians (30, 31). Research shows that social support and social esteem increase nurses’ mental resilience, which plays an important role in coping with stressors in the workplace (32) and increases nurses’ self-efficacy (33). The stress and coping theory provides a framework for understanding the role of social support in mental health (34). According to this theory, social support influences how individuals perceive and manage stress. Perceived stress, shaped by how people appraise situations and evaluate their coping abilities, strongly correlates with mental health outcomes such as anxiety, depression, and maladaptive behaviors (35). A study that identified social support as an external factor mediating the relationship between occupational stress related to COVID-19 and symptoms of anxiety and depression, demonstrated that higher levels of social support were associated with better mental health outcomes among primary care professionals (16). While there is extensive literature on the impact of the COVID-19 pandemic on anxiety, depression, and psychological resilience among nurses, few studies have been conducted in the context of primary care (16, 36, 37).

A study by Feng et al. (38) found that perceived social support was the third most important factor in protecting nursing staff from psychological stress. Social support has a positive impact on the relationship between stress levels and turnover intentions among medical staff at primary care centres (39) and nurses in hospitals (40, 41). These findings highlight the critical role of social support, or lack of it, as a factor that exacerbated staff burnout during the COVID-19 pandemic (42).

In order to address the gaps in the literature, our study aimed to assess the prevalence of anxiety and depressive symptoms, as well as analyze perceived social support among nurse practitioners in primary care.

Based on these considerations, the following research questions were formulated: (1) What is the prevalence of anxiety and depressive symptoms among nursing staff working in primary care (2) What is the level of perceived social support among this group? (3) Are there associations between sociodemographic or professional factors and nurses’ mental health or perceived social support?

## 2 Materials and methods

### 2.1 Study design and setting

This cross-sectional study was conducted between July 2022 and January 2023 among nurses employed in a network of private outpatient primary care clinics, after the COVID-19 epidemic restrictions were lifted in Poland. The study involved a homogeneous group of nurses working in clinics belonging to the same private healthcare organization, which provides services to patients across the country. All participants work under the same guidelines within structures governed by a standardized management model, which significantly reduces environmental variability. Their job positions are clearly defined, and their responsibilities are precisely outlined, ensuring a high degree of comparability in professional experience. Furthermore, for each respondent, this was their primary place of employment, meaning that their main professional activity is concentrated in this setting.

The study consisted of the following stages within the research project: (1) development of an electronic version of the questionnaire using Microsoft Forms, (2) distribution of the questionnaire link via email to nurses employed in outpatient clinics of the private healthcare provider, (3) statistical analysis of the collected data, and (4) preparation of the publication manuscript.

The study was approved by the Ethics Committee of the Medical University of Warsaw (registration number AKBE/98/2022).

### 2.2 Sampling and sample size

The estimated sample size was calculated based on the number of nurses for whom the outpatient clinic was their primary place of employment (*n* = 299), amounting to 168. The sample size was determined assuming a 95% confidence level, a 5% maximum error, and a population proportion of 0.5.

Out of a group of 299 nursing staff employees in 40 outpatient clinics of a private healthcare group, 199 participated in the study, accounting for 62.8% of the total. Data from 175 participants were analysed, while data from 24 questionnaires were excluded due to numerous omissions.

### 2.3 Data collection instruments

#### 2.3.1 Sociodemographic variables

The original questionnaire included a section on sociodemographic characteristics of the study group, as well as professional-related variables (education, speciality, number of workplaces, place of employment, management). Multiple regression analysis was used to develop models that explain socio-demographic and work-related variables of perceived social support, as measured by the MSPSS scale. The following variables were employed: dichotomous variables (gender, relationship status, and managerial position) and continuous variables (age and number of workplaces). The models were adjusted for anxiety and depression scores as measured by the HADS-M scale.

#### 2.3.2 Multidimensional Scale of Perceived Social Support (MSPSS)

To measure perceived social support, we used a Polish adaptation of the Multidimensional Scale of Perceived Social Support (MSPSS) (43), based on the original study by Zimet et al. (44). Use of the scale in the Polish language version is in accordance with the CC-BY 4.0 license.

This tool provides a quick overview of the perceived availability of support networks and assesses the sources that respondents consider most useful and valuable. The MSPSS takes into account three basic sources of support: significant others, family, and friends. The scale consists of 12 statements that respondents rate on a seven-point Likert scale, where 1 means “strongly disagree” and 7 means “strongly agree.” The higher the overall and subscale scores obtained by the participants, the greater the perceived social support (43).

In this study, the internal consistency of the MSPSS was *α* = 0.96 for the total score, α = 0.98 for friends, α = 0.96 for family, and α = 0.93 for significant others.

#### 2.3.3 The Hospital Anxiety and Depression Scale (HADS-M)

To assess the mental well-being of the nursing staff, a modified version of the Hospital Anxiety and Depression Scale (HADS) was used, based on the original study by Zigmont and Snaith (45) and consisting of two subscales: anxiety and depression. Permission to use the Polish adaptation of the HADS-M scale was obtained from its authors. The Polish version of the scale, known as HADS-M, includes an additional “irritability” subscale (46). The HADS-M scale used in the study comprises a total of 16 items, classified into three subscales: anxiety (7 items), depression (7 items) and irritability (2 items). Each response is scored from 0 to 3 points, with a maximum total score of 48 points. The higher score on a given subscale indicates a higher level of the assessed trait. Respondents were classified as follows for anxiety and depression: no disorder (0–7 points), borderline status (8–10 points), or disorder (11–21 points). For the irritability subscale, the classification was as follows: no disorder (0–2 points), borderline status (3 points), and disorder (4–6 points).

The internal consistency of the HADS-M was *α* = 0.94 for the total score, α = 0.87 for the anxiety subscale, α = 0.87 for the depression subscale, and α = 0.84 for the irritability subscale.

### 2.4 Data collection

The questionnaire was completed online via computer-assisted web interviewing (CAWI). The questionnaire was distributed via the intranet to all nurses employed in 40 outpatient clinics belonging to a private healthcare provider. Participants were informed about the purpose of the study and asked to provide informed consent. The following inclusion criteria had to be met to qualify for participation: (1) consent to participate in the study, (2) current employment as a nurse, (3) an outpatient clinic as the primary place of employment, and (4) at least 1 year of experience in outpatient care. All respondents provided informed consent prior to completing the questionnaire and were assured of its anonymity. Respondents were free to withdraw from the study at any time while completing the questionnaire.

### 2.5 Data analysis

The analysis was performed using Statistica 13.3 software with a statistical significance level of 0.05. The Shapiro–Wilk test was used to examine data distribution. Due to the lack of normal distribution and the different group sizes, nonparametric tests were used for the analysis. Spearman’s R test and Tau Kendall test were used to examine correlations. The Mann–Whitney U test was used to compare quantitative data. Multiple regression analysis was used to develop models that explain socio-demographic and work-related variables of perceived social support, as measured by the MSPSS scale. The following variables were employed: dichotomous variables (gender, relationship status, and managerial position) and continuous variables (age and number of workplaces).

The study involved 175 respondents, of whom 92% were women and 8% were men. The mean age was 38.60 ± 8.68 years. The participants had an average of 15.98 ± 9.21 years of total professional experience and 8.24 ± 5.94 years of experience in outpatient care. Most respondents (72%) were in a relationship. Over half of the respondents (57.72%) held a master’s degree, and one-third (33.71%) had a bachelor’s degree. A nursing specialty had been completed by 45.14% of the respondents. Employment at a single workplace was reported by 51.43% of the respondents, while 7.42% worked in a rural setting. In total, 17.86% of the respondents both managed a nursing team and worked as nurses (Supplementary Table S1).

## 3 Results

### 3.1 Perceived social support

The study participants scored 69.75 ± 13.08 points on the MSPSS scale. The nursing staff rated the support they received from significant others the highest (23.60 ± 4.71) (Table 1). Data analysis revealed negative, statistically significant correlations between age and support from significant others (rho = −0.156, *p* = 0.039), and between number of jobs and perceived support from family (*r* = −0.195, *p* = 0.010). Older respondents reported lower levels of support received from significant others. Similarly, respondents who reported holding multiple jobs rated the support received from their family as lower. Overall perceived support was positively correlated with a larger town/city where nurses were employed (rho = 0.193; *p* = 0.010), as well as with support from family (rho = 0.217; *p* = 0.004) and significant others (rho = 0.246; *p* = 0.001) (Table 2). There were no statistically significant differences in the assessment of overall social support or individual sources of support depending on gender (*p* = 0.111) or marital status (*p* = 0.260). However, management staff scored significantly higher in perceived overall social support (Z = 2.03, *p* = 0.042) and support from significant others (Z = 2.63, *p* = 0.008) (Table 3).

**Table 1:** Descriptive statistics for perceived social support (MSPSS) and anxiety, depression and irritability as measured by the HADS-M scale – descriptive statistics.

MSPSS	M (SD)	Med. (Min.–Max.)	Q1	Q3
Total	69.75 ± 13.08	72.00 (12–84)	62.00	82.00
Friends	22.91 ± 5.04	24.00 (4–28)	20.00	28.00
Family	23.24 ± 5.04	24.00 (4–28)	20.00	28.00
Significant others	23.60 ± 4.71	24.00 (4–28)	21.00	28.00

HADS-M	M (SD)	Med (Min.–Max.)	Q1	Q3
Total	15.54 ± 9.02	14.0 (0–41)	9.00	21.00
Anxiety	7.71 ± 4.52	7.0 (0–21)	4.00	10.00
Depression	4.57 ± 3.85	4.0 (0–16)	1.00	7.00
Irritability	3.26 ± 1.48	3.0 (0–6)	2.00	4.00

M, Mean; SD, Standard deviation; Med., Median; Min., Minimum; Max., Maximum; Q1, the first quartile; Q3, the third quartile.

**Table 2:** Correlations between age, work experience, education, number of jobs and perceived social support and HADS-M scores (*n* = 175).

Variable	MSPSS								HADS-M							
	Total		Friends		Family		Significant others		Total		Anxiety		Depression		Irritability	
	rho	*p*	rho	*p*	rho	*p*	rho	*p*	rho	*p*	rho	*p*	rho	*p*	rho	*p*
Age	−0.088	0.249	−0.075	0.338	−0.041	0.589	−0.156	0.602	−0.010	0.892	−0.052	0.495	0.012	0.874	0.040	0.602
Work experience	−0.060	0.432	−0.060	0.431	−0.008	0.920	−0.129	0.968	−0.030	0.697	−0.076	0.319	0.007	0.931	0.001	0.968
Work experience in an outpatient clinic	−0.249	0.804	−0.630	0.530	0.688	0.493	−1.469	0.236	0.012	0.872	0.012	0.874	−0.011	0.881	0.090	0.236
Education*	0.071	0.164	0.072	0.173	0.106	0.038*	0.051	0.232	0.042	0.406	0.087	0.088	−0.012	0.814	0.061	0.232
Place of residence**	0.151	0.003*	0.122	0.017	0.176	<0.001*	0.193	0.889	−0.035	0.495	−0.033	0.586	−0.028	0.586	−0.007	0.889
Number of jobs**	−0.104	0.041*	−0.044	0.391	−0.176	<0.001*	−0.074	0.011*	−0.040	0.433	−0.035	0.486	−0.013	0.799	−0.129	0.011*

rho, Spearman’s correlation coefficients; *p*, statistical significance, *Indicates *p* < 0.05, **Tau Kendall test.

**Table 3:** Comparison of perceived social support and HADS-M scale results in groups across marital status, gender, and professional roles (*n* = 175).

MSPSS	Med. (Min.–Max.)	Q1	Q3	*Z*	*p*
Total					
In a relationship				1.13	0.260
Yes (*n* = 126)	72 (12–84)	63.00	82.00		
No (*n* = 49)	71 (15–84)	61.00	75.00		
Gender				−1.59	0.111
Female (*n* = 161)	72 (12–84)	62.00	81.00		
Male (*n* = 14)	76 (60–84)	67.00	84.00		
Management				2.03	0.042*
Yes (*n* = 30)	79 (39–84)	67.00	84.00		
No (*n* = 145)	72 (12–84)	62.00	79.00		
Friends					
In a relationship				0.23	0.820
Yes (*n* = 126)	24 (4–28)	20.00	28.00		
No (*n* = 49)	24 (4–28)	20.00	28.00		
Gender				−1.51	0.132
Female (*n* = 161)	24 (4–28)	20.00	28.00		
Male (*n* = 14)	25.5 (20–28)	24.00	28.00		
Management				1.93	0.053
Yes (*n* = 30)	26 (6–28)	21.00	28.00		
No (*n* = 145)	24 (4–28)	20.00	28.00		
Family					
In a relationship				1.38	0.159
Yes (*n* = 126)	24 (4–28)	22.00	28.00		
No (*n* = 49)	24 (4–28)	20.00	28.00		
Gender				−1.41	0.167
Female (*n* = 161)	24 (4–28)	20.00	28.00		
Male (*n* = 14)	24.5 (20–28)	24.00	28.00		
Management				1.90	0.057
Yes (*n* = 30)	27 (11–28)	23.00	28.00		
No (*n* = 145)	24 (4–28)	20.00	28.00		
Significant others					
In a relationship				1.02	0.309
Yes (*n* = 126)	24 (4–28)	22.00	28.00		
No (*n* = 49)	24 (7–28)	20.00	28.00		
Gender				−1.08	0.282
Female (*n* = 161)	24 (4–28)	21.00	28.00		
Male (*n* = 14)	25 (20–28)	23.00	28.00		
Management				2.63	0.008*
Yes (*n* = 30)	27 (11–28)	24.00	28.00		
No (*n* = 145)	24 (4–28)	21.00	28.00		

HADS-M	Med. (Min.-Max.)	Q1	Q3	*Z*	*p*
Total					
In a relationship				1.00	0.315
Yes (*n* = 126)	14 (0–36)	9.00	22.00		
No (*n* = 49)	15 (1–36)	6.00	20.00		
Gender				1.70	0.090
Female (*n* = 161)	15 (0–36)	9.00	22.00		
Male (*n* = 14)	9 (2–36)	8.00	13.00		
Management				1.04	0.299
Yes (*n* = 30)	15 (3–36)	8.00	27.00		
No (*n* = 145)	14 (0–36)	9.00	20.00		
Anxiety					
In a relationship				1.04	0.301
Yes (*n* = 126)	7 (0–19)	5.00	10.00		
No (*n* = 49)	7 (0–21)	3.00	9.00		
Gender				1.71	0.087
Female (*n* = 161)	8 (0–21)	5,00	10.00		
Male (*n* = 14)	5.5 (0–17)	3,00	7.00		
Management				1.074	0.285
Yes (*n* = 30)	7.5 (2–18)	4.00	14.00		
No (*n* = 145)	7 (0–21)	5.00	9.00		
Depression					
In a relationship				1.08	0.282
Yes (*n* = 126)	4 (0–16)	1.00	7.00		
No (*n* = 49)	4 (0–15)	1.00	7.00		
Gender				1.28	0.202
Female (*n* = 161)	4 (0–16)	1.00	7.00		
Male (*n* = 14)	2 (0–13)	1.00	5.00		
Management				0.28	0.784
Yes (*n* = 30)	4 (0–14)	1.00	9.00		
No (*n* = 145)	4 (0–16)	1.00	7.00		
Irritability					
In a relationship				0.83	0.406
Yes (*n* = 126)	3 (0–6)	2.00	4.00		
No (*n* = 49)	3 (0–6)	2.00	4.00		
Gender				1.26	0.206
Female (*n* = 161)	3 (0–6)	2.00	4.00		
Male (*n* = 14)	2.5 (0–6)	2.00	4.00		
Management				2.57	0.012*
Yes (*n* = 30)	4 (1–6)	3.00	5.00		
No (*n* = 145)	3 (0–6)	2.00	4.00		

Med., Median, Min., Minimum, Max., Maximum, *Z*, *Z* value for the Mann–Whitney test; *p*, statistical significance; Q1, the first quartile; Q3, the third quartile. *Indicates *p* < 0.05.

### 3.2 Anxiety, depression and irritability

**Figure 1:**
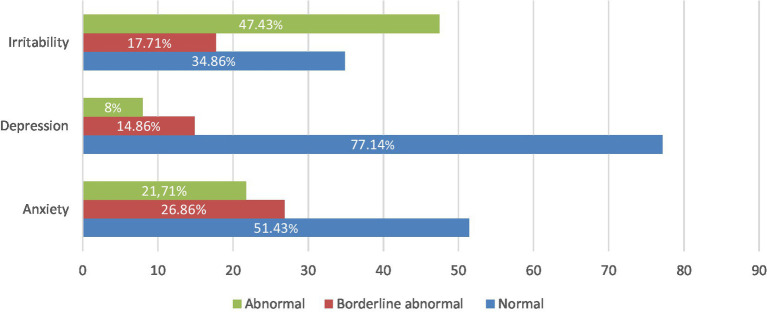
Mental well-being of respondents on the HADS-M scale.

In total, the respondents scored 15.54 ± 9.02 points on the HADS-M scale (Table 1). Anxiety disorders were identified in 21.71% of the respondents, depression in 8% of the respondents, and irritability in 47.43% of the nursing staff (Figure 1). There were no statistically significant correlations between the age of the respondents (rho = −0.010; *p* = 0.892), work experience in nursing (rho = −0.030; *p* = 0.697) and in the outpatient clinic (rho = 0.012; *p* = 0.872), education (rho = 0.056; *p* = 0.463), place of residence (rho = −0.042; *p* = 0.583) and number of jobs (rho = −0.029; *p* = 0.705) (Table 2). Irritability was only observed in relation to job position (Z = 2.57, *p* = 0.012), with this trait predominantly present among management personnel (Table 3).

### 3.3 Nurses’ mental well-being and perceived social support

Data analysis revealed a statistically significant negative correlation between total HADS-M and MSPSS scores (*r* = −0.401; *p* < 0.001). Additionally, statistically significant negative correlations were found between HADS-M subscale scores and perceived support from individual sources (*p* < 0.05). Respondents with lower scores for mental health symptoms (indicating better mental well-being) reported higher levels of perceived social support (Table 4). We used multiple regression to build models explaining the sociodemographic and work related variables of perceived social support measuring by MSPSS scale. Managerial positions were found to significantly increase the total score (*p* = 0.041), the score for friends (*p* = 0.041), and the score for significant others (*p* = 0.040). A statistically significant decrease in perceived social support was observed in the total score (*p* = 0.020) and the significant others score (*p* = 0.005), as well as in the number of workplaces (*p* = 0.004) and the family score (*p* = 0.004). The relationship status variable was insignificant in all models (*p* > 0.05). Details are presented in Table 5.

**Table 4:** Correlation between the HADS-M and MSPSS scales and subscales.

HADS-M	MSPSS – Total		MSPSS – Friends		MSPSS – Family		MSPSS – Significant other	
	rho	*p*	rho	*p*	rho	*p*	rho	*p*
Total	−0.401	<0.001*	−0.383	<0.001*	−0.366	<0.001*	−0.338	<0.001*
Anxiety	−0.384	<0.001*	−0.350	<0.001*	−0.343	<0.001*	−0.327	<0.001*
Depression	−0.405	<0.001*	−0.395	<0.001*	−0.376	<0.001*	−0.334	<0.001*
Irritability	−0.230	0.002*	−0.248	<0.001*	−0.208	0.006*	−0.190	0.011*

rho, Spearman’s correlation coefficients; *p*, statistical significance. *Indicates *p* < 0.05.

**Table 5:** The impact of socio-geographic and work-related data, as well as anxiety and depression, on perceived social support.

Variable	*B*	Standard error	*T*	95% Conf. interval		*p*
Model 1: MSPSS Total						
	***p* < 0.001**, *R*-squared = 0.213, MSE = 23.467					
Gender	5.68	3.52	1.61	0.109	−1.27	0.109
Age	−0.25	0.11	−2.34	0.020	−0.47	0.020*
In a relationship	3.39	2.11	1.61	0.110	−0.78	0.110
Number of Jobs	−2.67	1.46	−1.83	0.069	−5.54	0.069
Management	5.17	2.51	2.06	0.041	0.22	0.041*
HADS-M anxiety	−0.46	0.37	−1.25	−1.18	0.27	0.213
HADS-M depression	−0.78	0.40	−1.93	−1.57	0.02	0.055
Model 2: MSPSS Friends						
	***p* < 0.001**, *R*-squared = 0.142, MSE = 20.445					
Gender	1.83	1.43	1.27	−1.00	4.65	0.204
Age	−0.07	0.04	−1.53	−0.15	0.02	0.128
In a relationship	0.71	0.86	0.82	−0.99	2.40	0.412
Number of jobs	−0.33	0.59	−0.55	−1.50	0.84	0.584
Management	2.10	1.02	2.06	0.09	4.12	0.041*
HADS-M anxiety	−0.15	0.15	−1.01	−0.45	0.14	0.315
HADS-M depression	−0.27	0.16	−1.65	−0.59	0.05	0.101
Model 3: MSPSS Family						
	***p* < 0.001**, *R*-squared = 0.191, MSE = 18.695					
Gender	2.48	1.37	1.81	−0.23	5.18	0.073
Age	−0.07	0.04	−1.76	−0.16	0.01	0.080
In a relationship	1.44	0.82	1.75	−0.18	3.06	0.081
Number of jobs	−1.67	0.57	−2.95	−2.79	−0.55	0.004*
Management	1.18	0.98	1.20	−0.75	3.10	0.230
HADS-M anxiety	−0.11	0.14	−0.75	−0.39	0.17	0.454
HADS-M depression	−0.29	0.16	−1.87	−0.60	0.02	0.063
Model 4: MSPSS Significant other						
	***p* < 0.001,** *R*-squared = 0.019, MSE = 16.388					
Gender	1.38	1.28	1.07	−1.16	3.91	0.285
Age	−0.11	0.04	−2.84	−0.19	−0.03	0.005*
In a relationship	1.24	0.77	1.62	−0.27	2.76	0.108
Number of jobs	−0.67	0.53	−1.27	−1.72	0.37	0.206
Management	1.89	0.91	2.07	0.09	3.70	0.040*
HADS-M anxiety	−0.20	0.13	−1.50	−0.47	0.06	0.135
HADS-M depression	−0.21	0.15	−1.45	−0.50	0.08	0.149

B, regression coefficient; Standard Error, standard error; *T*-value, *t*-test, 95% Conf. interval, confidence intervals; *p*, statistical significance; *R*-squared, MSE, Mean square error. *Indicates *p* < 0.05.

## 4 Discussion

The findings showed that one-fifth of the respondents reported anxiety, 8% reported depression, and nearly half reported irritability. Previously, Halcomb et al. examined the mental health of primary care nursing staff during the pandemic using the Depression, Anxiety and Stress Scale (DASS-21). In their study, 39.6% of the participants reported depression, anxiety, or stress (47). The higher rates of anxiety and depression symptoms observed in the Halcomb et al. study compared to ours may be due to the use of a different assessment tool. However, timing may be the main determining factor, as the Halcomb et al. study was conducted in the pandemic.

As in the Halcomb et al. study, we attempted to assess the anxiety and depression of our participants. However, it should be noted that our findings are consistent with those of other researchers who have reported mental health symptoms among hospital staff during the pandemic (48, 49). These rates were particularly high, reaching approximately 30–50% of the staff. This problem affects more than just hospital staff. A study conducted in Japan among home health care professionals (home-HCWs) showed that they scored higher on depression than other medical groups, e.g., physicians (50). Similarly, a study in Turkey found that depression and anxiety rates were highest among hospital nurses (51.1 and 40.2%, respectively) and lowest among physicians (13.6 and 7.8%, respectively) of all medical professionals surveyed (51). In our study, nurses in managerial positions were significantly more irritable than nurses not holding such responsible positions (*p* = 0.285). These findings suggest that workload and associated fatigue are the main factors leading to psychological or psychiatric disorders. The work environment of primary care nurses differs from that of hospital nurses due to differences in organizational structure and the nature of occupational stressors. These differences may influence nurses’ experiences with stress, occupational burnout, and symptoms such as anxiety, depression, and irritability. Primary care nurses need extensive knowledge of various diseases and the skills necessary to provide care across multiple nursing domains. They establish long-term therapeutic relationships with patients and their families, work directly within the community, and are exposed more frequently to the various determinants of patient health. Their professional practice is also characterized by a higher degree of autonomy (52).

Research shows that social support reduces the psychological burden on healthcare professionals, including nurses, and helps them cope with stress (53–55). Our study confirmed that support from significant others is equally important for Polish nurses, though they rated perceived support from family and friends lower.

A Turkish study conducted before the outbreak of the COVID-19 pandemic showed hospital nurses to have moderate levels of social support, primarily from their families and, to a lesser extent, from significant others (32). Chinese (55) and Iranian (56) nurses working in hospitals during the pandemic reported slightly lower social support, although it was still at a moderate level. Shortly before the pandemic, Nowicki et al. assessed the relationship between perceived social support and nurses’ sense of security and life meaning. At that time, nurses ranked support from significant others as the most important (57). Culture is one of the variables that may influence how social support is provided, perceived, or received (58). People in more individualistic cultures tend to seek social support more often. For example, European Americans are more likely to rely on their social relationships, whereas Western Europeans may seek explicit assistance from family and friends when coping with stressful life events. Studies have shown that Asians are generally less inclined to rely on social support when dealing with stress (29, 58). The social and cultural context of social support in Poland reflects a combination of traditional values, such as the importance of family, and new challenges arising from an aging society. Key roles are also played by state social policy and support networks based on formal (institutional) and informal (family and friendship) interpersonal relationships. The study also found that older nurses reported lower levels of perceived social support (57). This is difficult to explain, though one might argue that years of routine work and experience in the profession reduce the need for support from others. However, it should be noted that these findings were primarily confirmed among nurses working in hospitals. There are no data on nurses employed in outpatient care.

Nevertheless, social support is important for the mental well-being of healthcare personnel (59). Although nurses provide support to patients, they also need support due to the nature of their work (60). In fact, it seems to be a universal truth that every human being needs some degree of support from others to function properly, especially for mental health. According to research, this is important for patients (61), nursing students (62), and active healthcare professionals. Research has shown that high levels of perceived social support contribute to resilience and well-being, which is consistent with our findings on mental well-being (63).

To sum up, our study highlights the importance of perceived social support for the well-being and mental health of nursing staff. Social support acts as a protective factor against the development of mental health disorders.

### 4.1 Limitations

This study has several limitations. Despite our efforts to refine the design, certain limitations were unavoidable. Firstly, as this was a cross-sectional study, no causal relationships can be inferred. Secondly, as participants were recruited from only one private-sector healthcare provider in Poland, the results may be difficult to generalize. Thirdly, as there were too few male nurses in our study, it is difficult to draw conclusions about whether gender is a factor associated with mental well-being and perceived social support in this group of nursing staff. Future research should assess larger populations recruited from public sector primary care facilities nationwide. This study assessed perceived social support using a scale that included three sources: significant others, family, and friends. However, this approach has a methodological limitation in that it does not include other potentially important sources of social support, such as professional networks, colleagues, social groups, and religious or sports organizations.

## 5 Conclusion

Perceived social support is an important protective factor for mental health. Nurses with higher mental well-being scores also reported higher levels of perceived social support. Nurses in managerial positions reported higher overall perceived social support and support from significant others than nurses providing direct patient care. A negative correlation was found between the age of nursing staff and perceived support from significant others, as well as between the number of workplaces and perceived support from family members.

The results of this study highlight the role of perceived social support as a protective factor for the mental health of nurses in primary care, particularly those with less professional experience and those in managerial roles. Efforts should be made to strengthen the role of significant others as sources of workplace support. Primary care nursing personnel should undergo periodic mental health assessments using validated screening tools. Organizational interventions that introduce improved work processes in primary care, arising from enhanced professional competencies and ongoing organizational changes in Poland, may effectively improve nurses’ mental health. The mental health of nurses in general practice has rarely been examined, and future studies should address this gap to inform initiatives that improve working conditions and promote psychological well-being in this group. As this was an exploratory study, the findings require confirmation in further research.

## Data Availability

The raw data supporting the conclusions of this article will be made available by the authors, without undue reservation.
