# Supplementary material for: Mental health and perceived social support among nurses working in primary care
Source: Front Public Health. 2025 Dec 4;13:1694797. doi: 10.3389/fpubh.2025.1694797 (PMC12711494; doi:10.3389/fpubh.2025.1694797)
Supplement: Supplementary file 1 [file Table_1.docx]

Supplementary Material Table 1S. Characteristics of the study group (n = 175)

| Variables |  | n (%) |
| --- | --- | --- |
| Gender | Female | 161 (92.00) |
|  | Male | 14 (8.00) |
| Education | Vocational secondary education | 8 (4.57) |
|  | Higher education, Bachelor’s degree | 59 (33.71) |
|  | Higher education, Master’s degree | 101 (57.72) |
|  | Higher education, PhD | 7 (4.00) |
| Marital status | Single | 36 (20.57) |
|  | Married/in a partnership | 126 (72.00) |
|  | Divorced/separated | 11 (6.29) |
|  | Widowed | 2 (1.14) |
| Specialty | Yes | 79 (45.14) |
|  | No | 88 (50.28) |
|  | In progress | 8 (4.57) |
| Place of employment | Village | 13 (7.42) |
|  | Small town (up to 20,000 inhabitants) | 13 (7.42) |
|  | Medium-sized town (> 20,000 to 100,000 inhabitants) | 24 (13.71) |
|  | Large town/ small city (> 100,000 to 300,000 inhabitants) | 23 (13.14) |
|  | Large city (> 300,000 inhabitants) | 35 (20.00) |
| Number of workplaces | 1 | 90 (51.43) |
|  | 2 | 67 (38.29) |
|  | 3 and more | 18 (10.28) |
| Management | Yes | 30 (17.86) |
|  | No | 145 (82.86) |
